# Regime-Aware Federated Aggregation (RAFA) for privacy-preserving energy load forecasting across heterogeneous European grid clients

**DOI:** 10.12688/openreseurope.24355.2

**Published:** 2026-08-06

**Authors:** Mahmoud Abbasi, Alfonso González Briones, Alesandro Gómez Villar, Javier Curto Hernández, Javier Prieto

**Affiliations:** 1 AIR Institute, Salamanca, Spain; 2 BISITE Research Group, University of Salamanca, Salamanca, Spain

**Keywords:** federated learning, energy load forecasting, distribution shift, regime detection, personalisation, Mahalanobis distance, ENTSO-E

## Abstract

**Background:**

Federated Learning (FL) enables collaborative model training across distributed clients without sharing raw data, making it attractive for privacy-sensitive energy forecasting. However, standard aggregation algorithms, most notably FedAvg and FedProx, assume stationary client data distributions, an assumption routinely violated in multi-national power grids where seasonal patterns, demand crises, and policy changes induce persistent regime shifts.

**Methods:**

We present RAFA (Regime-Aware Federated Aggregation), a novel FL framework that explicitly detects and adapts to distribution shifts in client model updates without accessing raw client data. RAFA comprises three components. These are a Mahalanobis-distance detector operating on random-projected update vectors; reliability-weighted aggregation that exponentially discounts shifted clients while preserving exact FedAvg behaviour when no shift is detected; and always-on personalised head fine-tuning that adapts each client’s final prediction layer to its local regime.

**Results:**

We validate RAFA on a new multi-national benchmark of six years (2019–2024) of hourly electrical load data for eight European bidding zones sourced from the ENTSO-E Transparency Platform. RAFA achieves a test MAE of 185.4 MW, an 11.8% improvement over FedAvg (210.2 MW), with by far the largest gain for the client exhibiting the strongest seasonal contrast (France, −30.5% ) and consistent improvements across the remaining clients (e.g., Portugal −7.6% , Poland −7.4% ). Ablation studies confirm both mechanisms contribute independently.

**Conclusions:**

RAFA provides a lightweight, privacy-preserving extension of standard federated aggregation that is robust to regime shifts in energy systems. The approach generalises to any federated setting with temporally non-stationary client distributions and a separable model architecture.

## Plain Language summary

Accurately forecasting electricity demand is essential for running power grids, but the underlying data is sensitive and cannot easily be shared between countries. *Federated learning* lets several grid operators jointly train a shared forecasting model while keeping their own data private. A problem is that electricity demand keeps changing through seasons, crises, and policy shifts, and standard federated methods, which assume stable conditions, lose accuracy when these “regime shifts” occur.

We introduce RAFA, a method that automatically detects when an operator’s data has shifted and adjusts its influence on the shared model accordingly, all without accessing any raw data. Tested on six years of real demand data from eight European countries, RAFA reduced forecasting error by about 12% compared with the most widely used federated method, with the biggest gains for countries whose demand varies most across seasons.

## 1. Introduction

Every hour, transmission system operators across Europe must balance supply against an uncertain demand, using load forecasts made minutes to hours in advance. A 100 MW forecast error cascades into reserve activation, balancing costs, and in extreme cases grid instability ( Abumohsen, Owda, and Owda 2023). As national grids grow more interconnected and data-driven, the case for collaborative forecasting that pools signal across borders has become strong; yet load data is commercially sensitive and, in many jurisdictions, legally restricted from crossing national boundaries.

Federated Learning (FL) ( McMahan et al. 2017) offers a natural answer. Participating clients train local models on their own data and share only model updates, never raw measurements, with a central server, which aggregates them into a global model. Keeping raw measurements on the client removes the most direct disclosure risk, which makes FL an attractive fit for cross-border energy forecasting where both commercial confidentiality and regulatory constraints are in play. It does not by itself make the exchange private. Model updates still carry information about the data that produced them, and gradient-inversion and membership-inference attacks exploit exactly that channel. We return to what our design does and does not protect in Section 4.7.

Standard FL algorithms, most notably FedAvg ( McMahan et al. 2017) and FedProx ( Li et al. 2020), were built on the implicit assumption that each client’s data distribution, however different from its peers, stays roughly stable over time. Energy systems rarely cooperate with this assumption. Demand patterns shift with seasons, economic cycles, and policy shocks. The 2022 European energy crisis reduced industrial consumption by 5–10% within months, permanently altering load profiles across the continent. We call these episodes *regime shifts*, persistent changes in the underlying load-generation pattern. When a subset of clients enters a new regime, their model updates pull the global model in a new direction, and naive averaging can corrupt a well-trained global model or derail convergence entirely.

In this paper, we present RAFA, a novel FL framework that explicitly detects and adapts to distribution shifts in client model updates. RAFA makes three principal contributions.

• **Mahalanobis regime detector.** A privacy-preserving detector that monitors the Mahalanobis distance of random-projected client update vectors with respect to a rolling reference distribution, flagging clients whose update behaviour has significantly diverged.
• **Reliability-weighted aggregation.** A soft aggregation rule that exponentially down-weights regime-shifted clients proportionally to their deviation, protecting the global model while preserving exact FedAvg behaviour when no shift is detected.
• **Always-on personalised head fine-tuning.** A decoupled personalisation mechanism that continuously adapts the final prediction head of each client’s model to its local regime, independent of whether a regime shift is detected, while keeping the shared base frozen for federation.

We validate RAFA on a novel multi-national benchmark comprising six years (2019–2024) of hourly actual electrical load data for eight European bidding zones, namely Netherlands (NL), Germany-Luxembourg (DE_LU), France (FR), Spain (ES), Portugal (PT), Belgium (BE), Austria (AT), and Poland (PL), sourced from the ENTSO-E Transparency Platform ( ENTSO-E 2024). Experiments demonstrate that RAFA improves forecasting accuracy over FedAvg, FedProx, and per-client local baselines, with by far the largest gain for the client exhibiting the strongest seasonal contrast (France, winter/summer ratio 1.48) and consistent improvements across the remaining clients.

### 1.1 Motivation for distribution shift in multi-national energy FL

To motivate the problem concretely, consider two clients in our benchmark, France (FR) and Spain (ES). FR has a winter-to-summer load ratio of 1.48, meaning winter demand is 48% higher than summer demand due to widespread electric heating, while ES exhibits a near-flat ratio of 1.02, reflecting a Mediterranean climate with minimal heating load. A global model trained by naive averaging must simultaneously represent both regimes, leading to systematic under- or over-fitting for both clients.

Furthermore, the 2022 European energy crisis introduced a structural regime shift across multiple clients simultaneously; reduced industrial activity, energy efficiency campaigns, and demand response programmes collectively lowered load levels by 5–10% relative to pre-crisis baselines ( International Energy Agency 2022). Standard FL algorithms have no mechanism to detect this kind of correlated shift or to adjust aggregation weights accordingly.

The remainder of this paper is structured as follows. Section 2 reviews related work in federated learning, non-IID adaptation, and energy load forecasting. Section 3 formally defines the federated energy forecasting problem and the regime shift model. Section 4 presents the RAFA framework. Section 5 describes the experimental setup. Section 6 presents results and ablation studies. Section 7 discusses limitations and future directions. Section 8 concludes.

## 2. Related work

The federated learning literature has expanded considerably since FedAvg, but much of that progress addresses a narrower problem than the one studied here. Methods for spatial heterogeneity, where clients have different but largely static feature or label distributions, are now reasonably mature. The harder and less-studied setting is temporal heterogeneity, where client distributions shift during training and the optimisation target moves over time. That gap motivates RAFA, and the following discussion examines how far existing work has gone toward closing it.

### 2.1 Federated learning: foundations and evolving challenges

McMahan et al. (2017) formalised FL with FedAvg, in which clients execute multiple local SGD steps and transmit model updates to a server that averages them weighted by dataset size. Despite its simplicity, FedAvg remains the dominant baseline because it decouples model training from raw-data sharing, enabling privacy-preserving collaboration at scale. Li et al. (2020) identified and formalised the mechanism behind its primary failure mode; when client data distributions diverge, local objectives pull updates in conflicting directions, a phenomenon they term *client drift*, causing the global model to converge to a region that is suboptimal for every participant. FedProx addresses this with a proximal term bounding the distance each local update can travel from the global reference point.

The heterogeneity problem has since been reframed as multi-dimensional. Jimenez G. et al. (2024) propose a taxonomy that partitions data heterogeneity into five axes, including quantity skew (unequal dataset sizes), feature skew (different input distributions), label skew (different class frequencies), temporal skew (client distributions shift over time), and concept skew (the same input maps to different labels across clients). The authors establish through meta-analysis that temporal skew is the least-studied axis despite being the most prevalent in longitudinal deployments such as energy monitoring. In parallel, Arbaoui et al. (2024) survey aggregation strategies across three design objectives, personalisation, optimisation, and robustness, and conclude that non-stationarity is the most critical open challenge, because neither personalised methods nor clustering-based approaches assume the distribution continues to evolve after an initial adaptation phase.

Clustered federated learning, which assigns clients to homogeneous groups before aggregation, has attracted particular interest as a practical response to spatial heterogeneity ( Ben Ali et al. 2025). Recent surveys find clustered methods consistently outperform global aggregation when client distributions differ statically. The key limitation is membership rigidity. Once a client is assigned to a cluster, the assignment persists, making the method blind to the within-client evolution that characterises seasonal energy systems.

An important emerging trend is the integration of pre-trained foundation models into federated pipelines. Hosseinalipour et al. (2025) introduce the concept of multi-modal, multi-task federated foundation models (FedFMs) for power grids, demonstrating that large pre-trained models can be fine-tuned across grid-edge clients in a privacy-preserving manner for tasks spanning load forecasting, anomaly detection, and demand response. This line of work underscores that the heterogeneity challenge is deepening, not resolving. As model capacity grows, the sensitivity to distributional mismatch between pre-training and deployment increases.

Byzantine robustness, addressed by Blanchard et al. (2017) and Yin et al. (2018), represents a structurally separate concern. Byzantine defences identify clients whose updates deviate maliciously from the consensus direction and suppress their influence. RAFA’s reliability weighting is superficially similar but operates from an opposite premise; large update deviations are treated as *informative* rather than malicious, signalling genuine regime change rather than adversarial intent.

### 2.2 Temporal Non-stationarity and concept drift in federated learning

The theoretical and practical difficulties of handling concept drift within a federated privacy model have been recognised since at least the survey of Casado et al. (2022), who document that virtually all established drift detectors, ADWIN, Page-Hinkley, DDM, KSWIN, require centralised access to a sliding data window, a requirement that is structurally incompatible with FL’s privacy model. Their conclusion is sobering, adapting any classical drift-detection procedure to the federated setting requires either weakening privacy guarantees, accepting substantial detection delays, or redesigning the detection mechanism from scratch.

Jothimurugesan et al. (2023) take the redesign path with FedDrift, which clusters clients by their estimated current posterior and reassigns them dynamically as distributions evolve. FedDrift requires a reference distribution per cluster and detects drift by comparing current client updates against that reference. A sound approach but one that assumes *synchronous* drift, all clients in a cluster change at the same time. In interconnected European electricity markets, where each bidding zone responds to its own demand shocks, weather regimes, and policy changes on independent schedules, this synchrony assumption is violated structurally.

A recent dedicated system is FLAME ( Mavromatis, De Feo, and Khan 2024), which monitors per-client output-distribution statistics and reconfigures the federation topology reactively when drift is detected. It is privacy-compatible, operates at the client level, and avoids the synchrony assumption. Its detection signal, however, is derived from model outputs, predictions or loss trajectories on local validation data. Output-level signals necessarily lag the underlying distributional shift; by the time a forecasting model’s accuracy degrades, the regime has already changed and several rounds of corrupted updates have been aggregated.

RAFA addresses this latency by detecting regime change in the geometry of weight-update vectors rather than in model outputs. Update geometry responds to distributional shift immediately, within the same round in which the shift begins, providing an early-warning signal that output-level monitors structurally cannot.

### 2.3 Deep learning for energy load forecasting

Short-term electricity load forecasting (STLF) is a safety-critical grid operation task. The classical toolkit, ARIMA-family models ( Box et al. 2015) for decomposing load into trend, seasonality, and residual, and exponential smoothing ( Holt 2004) as the operational forecasting workhorse, remains widely deployed because of its interpretability and low data requirements, but is known to underperform under non-stationary demand patterns.

Deep learning displaced statistical baselines in STLF benchmarks beginning around 2016. Dong et al. (2025) survey a decade of deep-learning-based STLF, tracing successive architectural waves; LSTMs for capturing temporal dependencies, CNNs for extracting local patterns, and attention-based models for long-range correlation, culminating in the Transformer as the dominant architecture family. Within Transformers, the iTransformer of Liu et al. (2024) reports leading accuracy on the multivariate benchmarks it evaluates by inverting the conventional token design making it particularly suited to the highly correlated, multivariate structure of cross-border energy load.

The architectural landscape continues to diversify rapidly. Kim et al. (2025) document a renaissance in time-series forecasting architecture, cataloguing the rise of hybrid models combining Transformers with Mamba state-space layers for long-context efficiency, diffusion-based probabilistic forecasters for uncertainty quantification, and large pre-trained time-series foundation models capable of zero-shot STLF. Critically, the survey identifies distribution shift as the most persistent unsolved challenge across all architectures, a finding that directly motivates the federated, regime-aware design of RAFA.

All of these methods, from classical ARIMA to the most recent foundation models, implicitly assume that training data can be pooled. For cross-border energy forecasting under GDPR and national grid-data confidentiality agreements, that assumption cannot be satisfied.

### 2.4 Federated learning for energy systems

The intersection of FL and power systems has grown substantially since 2024, driven by smart-meter rollouts, grid digitalisation, and tightening energy-data privacy regulations. Zhang et al. (2026) survey FL for smart grids, covering the full generation-to-consumption stack and documenting the principal vulnerabilities, such as poisoning, model inversion, and free-riding, that accompany FL deployment in critical infrastructure. Their analysis establishes that FL is no longer a theoretical proposal for energy systems. Production-grade FL systems are operating across all three grid stages, and the research agenda has shifted from feasibility to robustness and scalability.

At the forecasting layer, Rahman et al. (2025b) demonstrate personalised FL for residential load prediction over multi-hop AMI networks, developing a meta-learning aggregation strategy that jointly minimises forecast error and communication latency under heterogeneous device capacities. Rahman, Kumar, and Nguyen (2025a) extend this line of work to the MAML framework, showing that a single round of local fine-tuning on top of a collaboratively trained global model achieves better personalisation than either local-only or global training under realistic non-IID smart-meter distributions. Looking forward, Hosseinalipour et al. (2025) argue that the natural evolution of FL for grids is the adoption of federated foundation models. Large multi-task models pre-trained on heterogeneous grid data that can be fine-tuned at the edge for local forecasting, anomaly detection, and demand response simultaneously.

What is absent from all of this work is any mechanism to detect and respond to *within-round* distributional change. Every method above, whether aggregation-based, meta-learning-based, or foundation-model-based, trains a fixed-architecture model against a stationary (or slowly varying) data distribution. None detects the regime changes, such as energy-crisis demand spikes, post-lockdown recovery, sudden renewable integration, that fundamentally alter the forecasting task mid-deployment. This is precisely the gap that RAFA addresses.

### 2.5 Distance-based novelty and out-of-distribution detection

RAFA’s regime-shift detector draws on a mature body of work on distance-based OOD detection. The foundational contribution is from Lee et al. (2018), who showed that the class-conditional Mahalanobis distance in a DNN’s penultimate representation space is a highly effective, training-free OOD score. It outperformed softmax confidence and density-based baselines across a wide range of in-distribution and OOD dataset pairs, and can be computed post-hoc without modifying the trained model. The geometric intuition is that the covariance-normalised distance captures deviation relative to the learned representation geometry rather than in raw feature space, making it invariant to feature scaling and directional anisotropy. Ren et al. (2021) strengthened the method by subtracting a background-class baseline from the Mahalanobis score, substantially improving near-OOD detection, the setting where the distribution shift is small and progressive, precisely as in seasonal load-regime transitions.

Recent work has pushed the OOD detection frontier in two directions. Gao, He, and Pu (2025) combine feature-space, logit-space, and softmax-space information via an optimal transport scoring function (OTOD), outperforming the Mahalanobis baseline by substantial margins on standard benchmarks, demonstrating that geometric and transport-based cues are complementary. Claros Olivares et al. (2026) benchmark 30 post-hoc scoring functions across CNN and Vision Transformer encoders, and find that the *learned feature-space geometry* is the primary determinant of OOD efficacy; geometry-aware scores based on nearest-neighbour distances and prototype boundaries dominate under strong distributional shifts. Their setting is image classification rather than federated update vectors, so the result is suggestive rather than confirmatory here, but it motivates the design choice in RAFA; by operating on the update-vector geometry rather than the output space, the detector operates in the representation layer most sensitive to distributional change.

Computing a full Mahalanobis distance in the parameter space of a practical FL model is intractable; the parameter dimension typically exceeds $10^{6}$ . Johnson-Lindenstrauss random projections ( Johnson and Lindenstrauss 1984) resolve this by mapping update vectors to a low-dimensional subspace while provably preserving pairwise distances up to a (1±ϵ) factor with probability 1−δ for a projection dimension of $O\left(\epsilon^{-2}\log\left(1/\delta \right)\right)$ . The resulting projection-then-Mahalanobis detector scales to arbitrary model sizes, adds no communication overhead beyond the standard FL update, and requires no labelled drift examples or held-out reference data.

## 3. Background and problem formulation

Before describing RAFA, we need to be precise about two things, namely what the federation is jointly optimising, and what it means for a client’s data-generating process to shift in a way the server cannot observe directly. The notation introduced here carries through the rest of the paper.

### 3.1 Federated energy forecasting

Consider a federation of K clients $C=\left\{c_{1},\ldots ,c_{K}\right\}$ . Each client $c_{k}$ holds a private time series of hourly electrical load observations.

$$D_{k}=\left\{\left(x_{t}^{\left(k\right)},y_{t}^{\left(k\right)}\right):t=1,\ldots ,T_{k}\right\},$$

where $x_{t}^{\left(k\right)}\in {\mathbb{R}}^{d_{\text{in}}}$ is a feature vector comprising lagged load values and calendar features (see Section 5.1), and $y_{t}^{\left(k\right)}\in \mathbb{R}$ is the target load value at time t .

The goal is to collaboratively learn a shared global model $f_{\theta }:{\mathbb{R}}^{d_{\text{in}}}\to \mathbb{R}$ that minimises the weighted empirical risk:

$$F\left(\theta \right)=\sum_{k=1}^{K}p_{k}\;L_{k}\left(\theta \right),$$

where $L_{k}\left(\theta \right)=E_{\left(x,y\right)∼D_{k}}\left[\ell \left(f_{\theta }\left(x\right),y\right)\right]$ is the local loss (MSE), $p_{k}=n_{k}/n$ is the data fraction of client k , and $n=\sum_{k}n_{k}$ is the total number of samples.

### 3.2 Regime shift model

We denote the data distribution of client k at FL round r as $P_{k}^{\left(r\right)}$ . A *regime shift* at client k occurs at round r if the Kullback-Leibler divergence between the current and reference distributions exceeds a threshold if

$$\mathrm{KL}\;\left(P_{k}^{\left(r\right)}∥P_{k}^{\left(\mathrm{ref}\right)}\right)>\delta .$$

Because raw data is never shared, Equation (3) cannot be computed directly. Instead, RAFA detects regime shifts by monitoring the distribution of model update vectors, a proxy that is both privacy-preserving and sensitive to distribution changes, as shown empirically in Section 6.3. Table 1 summarises the notation used throughout the paper.

**Table 1.** Notation summary.

Symbol	Meaning	Symbol	Meaning
K	Number of clients	p	Total model parameters
r	Communication round index	$P\in {\mathbb{R}}^{d\times p}$	Fixed random projection matrix
R	Total communication rounds	$\mu_{r},\Sigma_{r}$	Reference mean and covariance
E	Local training epochs per round	$D_{k}^{\left(r\right)}$	Mahalanobis distance of client k
$\theta_{r}$	Global model at round r	τ	Detection threshold (chi-squared quantile)
$\theta_{k}^{\left(r\right)}$	Client k ’s local model at round r	$S_{k}^{\left(r\right)}\in \left\{0,1\right\}$	Regime-shift flag for client k
$\Delta_{k}^{\left(r\right)}$	Model update $\theta_{k}^{\left(r\right)}-\theta_{r-1}$	$\alpha_{k}^{\left(r\right)}$	Reliability weight for client k
$p_{k}=n_{k}/n$	Data fraction of client k	${\hat{\alpha }}_{k}^{\left(r\right)}$	Normalised reliability weight
$z_{k}^{\left(r\right)}\in {\mathbb{R}}^{d}$	Random-projected update vector	β	Penalty sharpness hyperparameter
d	Projection dimension ( d≪p )	W	Reference window length (rounds)

## 4. Methodology for Regime-aware federated aggregation (RAFA)

RAFA starts from a direct observation, a client whose model updates diverge from the historical population is likely experiencing a distributional shift in its underlying data. Acting on that signal, without accessing raw data, is the central design challenge, and the three components below address it from complementary angles. The complete algorithm is presented in Section 4.4.

Figure 1 provides an overview of the full system. At each communication round, every client trains locally, computes a compact projected update vector $z_{k}$ (1 KB), and sends both its model parameters and $z_{k}$ to the server. The server maintains a rolling reference buffer of past projected vectors, uses it to detect regime-shifted clients via Mahalanobis distance, assigns reliability weights, and returns a weighted global model to all clients. Each client additionally fine-tunes a small personalised head locally, which is never transmitted.

**Figure 1.**
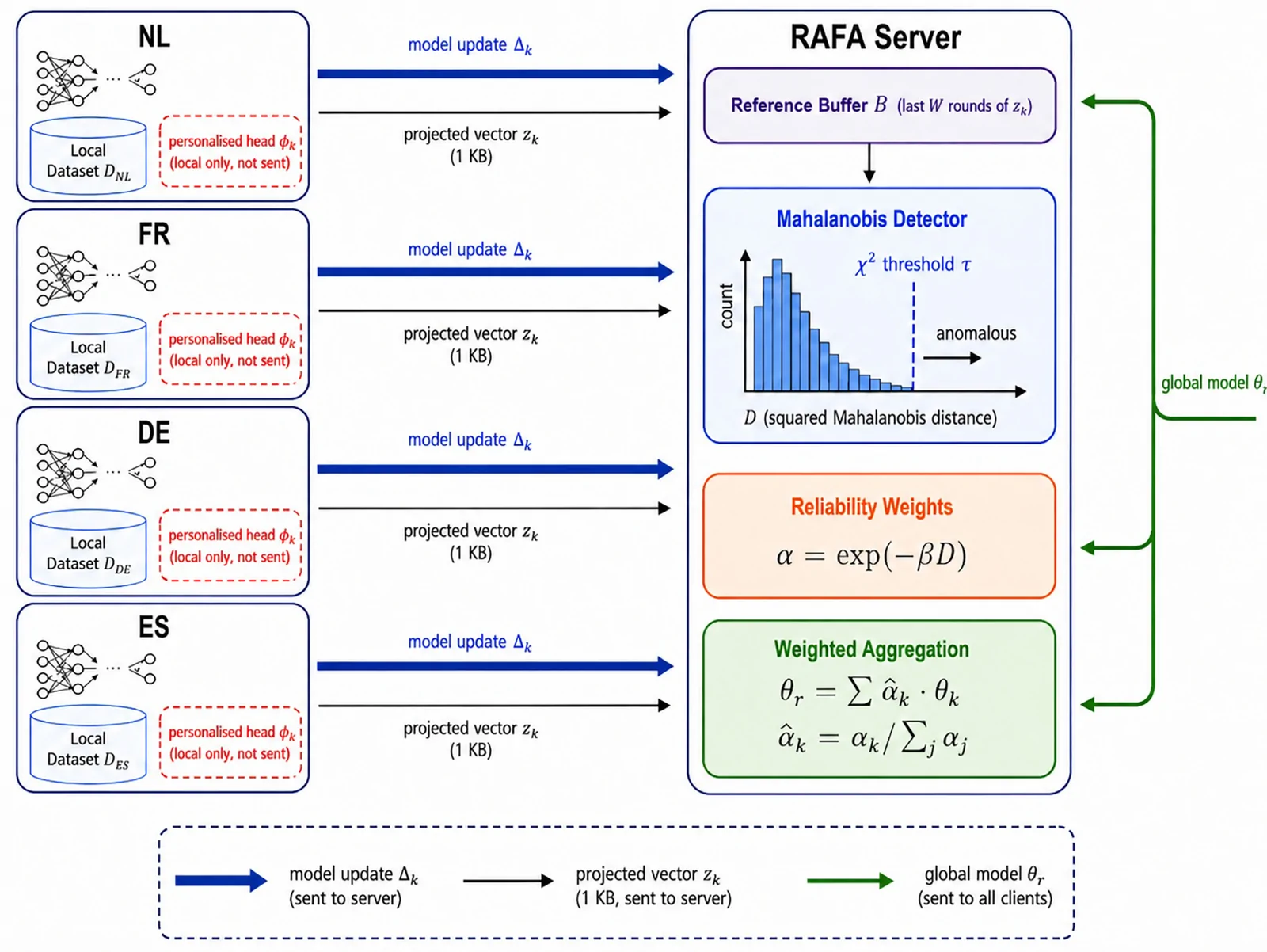
Overview of the RAFA framework. Each client sends model updates $\Delta_{k}$ and a compact projected vector $z_{k}$ (1 KB) directly to the server. The server detects regime-shifted clients via Mahalanobis distance against a rolling reference buffer B , assigns reliability weights $\alpha_{k}\propto p_{k}\exp\left(-\text{βmax}\left(0,D_{k}-\tau \right)\right)$ , and returns a reliability-weighted global model $\theta_{r}$ to all clients. Each client maintains a personalised head $\phi_{k}$ locally (red dashed box) that is never transmitted to the server.

### 4.1 Mahalanobis regime detector

At each round r , every client $c_{k}$ transmits a model update vector.

$$\Delta_{k}^{\left(r\right)}=\theta_{k}^{\left(r\right)}-\theta_{r-1},$$

where $\theta_{k}^{\left(r\right)}$ is the result of E local SGD steps on client k starting from the global model $\theta_{r-1}$ .

*Dimensionality Reduction via Random Projection*

For neural network models with p≫d parameters, computing the Mahalanobis distance in the full parameter space is intractable. We project update vectors into a low-dimensional space via a fixed random Gaussian projection matrix $P\in {\mathbb{R}}^{d\times p}$ :

$$z_{k}^{\left(r\right)}=P\cdot \frac{\Delta_{k}^{\left(r\right)}}{∥\Delta_{k}^{\left(r\right)}∥},$$

where the update is normalised to unit norm before projection to remove the trivial scale dependence on learning rate and number of local steps. Entries of P are i.i.d. N(0,1/d) , generated once at the server. By the Johnson-Lindenstrauss lemma ( Johnson and Lindenstrauss 1984), pairwise distances are preserved up to a (1±ε) factor with high probability.

*Reference distribution estimation*

The server maintains a rolling reference distribution over the last W rounds. The reference mean $\mu_{r}$ and diagonal variance $\sigma_{r}^{2}\in {\mathbb{R}}^{d}$ are updated at every round using all K clients.

$$\begin{array}{l} \mu_{r}=\frac{1}{\left|R_{W}\right|}\sum_{\left(k,s\right)\in R_{W}}z_{k}^{\left(s\right)},\\ \sigma_{r}^{2}=\frac{1}{\left|R_{W}\right|}\sum_{\left(k,s\right)\in R_{W}}{\left(z_{k}^{\left(s\right)}-\mu_{r}\right)}^{2}+\varepsilon 1, \end{array}$$

where $R_{W}=\left\{\left(k,s\right):s\in \left[r-W,r-1\right]\right\}$ is the window of recent client-round pairs and $\varepsilon =10^{-6}$ is a regularisation constant. The diagonal approximation reduces memory from $O\left(d^{2}\right)$ to O(d) and is motivated by the approximate independence of projected components under random Gaussian projections ( Johnson and Lindenstrauss 1984).

*Distance computation and thresholding*

The Mahalanobis distance of client k ’s update at round r with respect to the reference is given by

$$D_{k}^{\left(r\right)}=\sqrt{{\left(z_{k}^{\left(r\right)}-\mu_{r}\right)}^{⊤}\mathrm{diag}\;{\left(\sigma_{r}^{2}\right)}^{-1}\left(z_{k}^{\left(r\right)}-\mu_{r}\right)}.$$

A client is flagged as regime-shifted if its distance exceeds threshold τ, namely

$$S_{k}^{\left(r\right)}=1\;\left[D_{k}^{\left(r\right)}>\tau \right].$$

The threshold τ is set as the (1−α) -quantile of the chi-squared distribution with d degrees of freedom.

$$\tau =\sqrt{\chi_{d,1-\alpha }^{2}},$$

where α=0.05 is the target false-positive rate. Under the assumption that $z_{k}^{\left(r\right)}$ follows a multivariate normal with diagonal covariance $\sigma^{2}I$ , the squared Mahalanobis distance follows a $\chi_{d}^{2}$ distribution, giving τ=17.156 for d=256 .

The chi-squared threshold above is an approximation, and it is worth being explicit about where it is loose. The result holds for a known mean and covariance and for independent Gaussian components, and neither condition holds exactly here. The reference statistics are estimated from at most W·K = 80 client-round observations in d = 256 dimensions, so the per-component variances are estimated from fewer samples than there are dimensions and are biased downward, which inflates the distance. Normalising the update to unit norm before projection also induces a weak negative dependence among the components. The nominal false-positive rate should therefore be read as a scale-setting convention rather than a calibrated guarantee, and τ is best understood as a fixed operating point whose empirical behaviour we report in Section 6.3. We also evaluate an empirically calibrated alternative, with τ set to the (1-α) quantile of the distances observed during warm-up, in Section 6.8.

Because the reference window pools observations over all clients as well as over rounds, it describes the recent update behaviour of the federation as a whole. A large distance therefore means that a client's update is atypical relative to the current cross-client population, which mixes two distinct effects: a client whose own data-generating process has changed, and a client that is simply persistently unlike its peers. Separating them calls for a per-client reference buffer, in which each client is compared against its own history. We keep the pooled form as the default because it is what the published version used, and report the per-client variant as an ablation in Section 6.8.

*Warm-up phase*

Detection is suppressed for the first W rounds while the reference window accumulates sufficient observations. Suppression applies both to the shift flags and to the reliability weights of Section 4.2: during warm-up every client is aggregated with its data-fraction weight, exactly as in FedAvg. This matters because the downward bias in the variance estimate is largest precisely when the buffer is nearly empty, so distances are most inflated in the rounds where they are least meaningful. The implementation accompanying the first version of this article suppressed the flags but still applied the penalty during warm-up; we report the consequences of that discrepancy in Section 6.7.

### 4.2 Reliability-weighted aggregation

Standard FedAvg aggregates client models according to

$$\theta_{r}=\sum_{k=1}^{K}p_{k}\;\theta_{k}^{\left(r\right)}.$$

RAFA replaces the data-fraction weights $p_{k}$ with reliability weights $\alpha_{k}^{\left(r\right)}$ that discount regime-shifted clients.

$$\alpha_{k}^{\left(r\right)}=p_{k}\cdot g\;\left(D_{k}^{\left(r\right)}\right),$$

where $g:{\mathbb{R}}_{+}\to \left(0,1\right)$ is a monotonically decreasing penalty function. We use a soft-threshold exponential function.

g(d)=exp(−β·max(0,d−τ)),

with hyperparameter β>0 controlling penalty sharpness. For d≤τ (non-shifted clients), g(d)=1 and weights reduce to standard FedAvg. Weights are normalised to sum to one.

$${\hat{\alpha }}_{k}^{\left(r\right)}=\frac{\alpha_{k}^{\left(r\right)}}{\sum_{j=1}^{K}\alpha_{j}^{\left(r\right)}},$$

This gives the global update rule

$$\theta_{r}=\sum_{k=1}^{K}{\hat{\alpha }}_{k}^{\left(r\right)}\;\theta_{k}^{\left(r\right)}.$$

This formulation has two key properties. (i) it reduces to exact FedAvg when no client is regime-shifted ( $D_{k}^{\left(r\right)}\leq \tau$ for all k ); and (ii) a highly shifted client contributes negligible weight, protecting the global model from corrupted updates.

Property (i) deserves emphasis because it bounds how much this component can do. The penalty depends on the continuous distance through max(0, D - τ), and is identically one whenever no client exceeds τ. In rounds where nothing is flagged the reliability weights equal the data-fraction weights, so the aggregation step is not merely similar to FedAvg but arithmetically identical to it. Any accuracy difference attributed to reliability weighting must come from the rounds in which at least one client is flagged, and its size is bounded by how far the weights move in those rounds. We report both quantities in Section 6.6 rather than leaving the mechanism's contribution to be inferred.

The binary shift flag plays no part in the weighting. It is reported as a diagnostic and used to delimit the warm-up behaviour described above; the aggregation is driven entirely by the continuous distance.

### 4.3 Always-on personalised head fine-tuning

After local training, each client fine-tunes only the final prediction layer (head) of its model copy at learning rate ρ=η/5 for $E_{p}$ additional epochs, with all base layers frozen:

$$\phi_{k}^{\left(r\right)}=\arg\min_{\phi }\;L_{k}\;\left(f_{\left[\theta_{k}^{\left(r\right)},\phi \right]};D_{k}\right),$$

where $\left[\theta_{k}^{\left(r\right)},\phi \right]$ denotes the model with base weights $\theta_{k}^{\left(r\right)}$ and head weights ϕ .

The personalised head $\phi_{k}^{\left(r\right)}$ is not transmitted to the server; it remains local, preserving the privacy of regime-specific adaptation. The server aggregates only the base parameters $\theta_{k}^{\left(r\right)}$ . Evaluation uses the personalised model $f_{\left[\theta_{k}^{\left(r\right)},\phi_{k}^{\left(r\right)}\right]}$ .

That sentence carries more weight than it appears to, and the first version of this article did not draw out its consequence. The model a client reports with is its locally trained base plus a fine-tuned head, whereas a plain FedAvg client reports with the aggregated global model. Scoring one against the other compares two different objects, and any resulting difference confounds the aggregation rule with the presence of local adaptation. Section 5.4 therefore defines three evaluation protocols and reports every method under all three, so that each comparison isolates one mechanism at a time.

This decoupled design separates two concerns. Aggregation (what the server sees) is controlled by the detector via reliability weights; prediction (what each client reports) always benefits from local adaptation regardless of whether a regime shift is detected. The personalisation component is deliberately conventional, and its relationship to existing work is worth stating plainly rather than claiming as novel. FedPer ( Arivazhagan et al. 2019) splits the network into shared base layers and personalisation layers, federating the former and keeping the latter local permanently. LG-FedAvg ( Liang et al. 2020) inverts the split. Our head fine-tuning is, mechanically, FedPer with a one-layer personalisation block re-fitted from the current base each round. The distinction that matters here is not the personalisation scheme but what it is combined with: those methods leave the aggregation rule untouched, whereas the same head here sits on an aggregation rule that responds to a shift signal. Because that is the only structural difference, a FedAvg variant carrying an identical head is the right control for isolating regime-aware aggregation, and we report it throughout Section 6.

### 4.4 Complete algorithm

Figure 2 collects all three RAFA components, Mahalanobis regime detection, reliability-weighted aggregation, and personalised head fine-tuning, into a single, self-contained procedure. The algorithm operates in two phases. During the *warm-up phase* (rounds r≤W ), the server accumulates projected update vectors into the reference buffer B but has not yet seen enough history to distinguish normal variation from a genuine regime shift; detection flags are therefore suppressed to avoid unstable early-round decisions while the reference distribution is still forming. Once the buffer is full ( r>W ), the *detection phase* begins; at every round the server computes the reference mean and covariance from the most recent W rounds, scores each client’s current update via Mahalanobis distance, and derives reliability weights that soft-discount any client flagged as shifted. Meanwhile, each client fine-tunes its personalised head locally every round, regardless of phase; the head is never transmitted, so it adds no communication overhead. The complete round-by-round flow is given in Figure 2.

**Figure 2.**
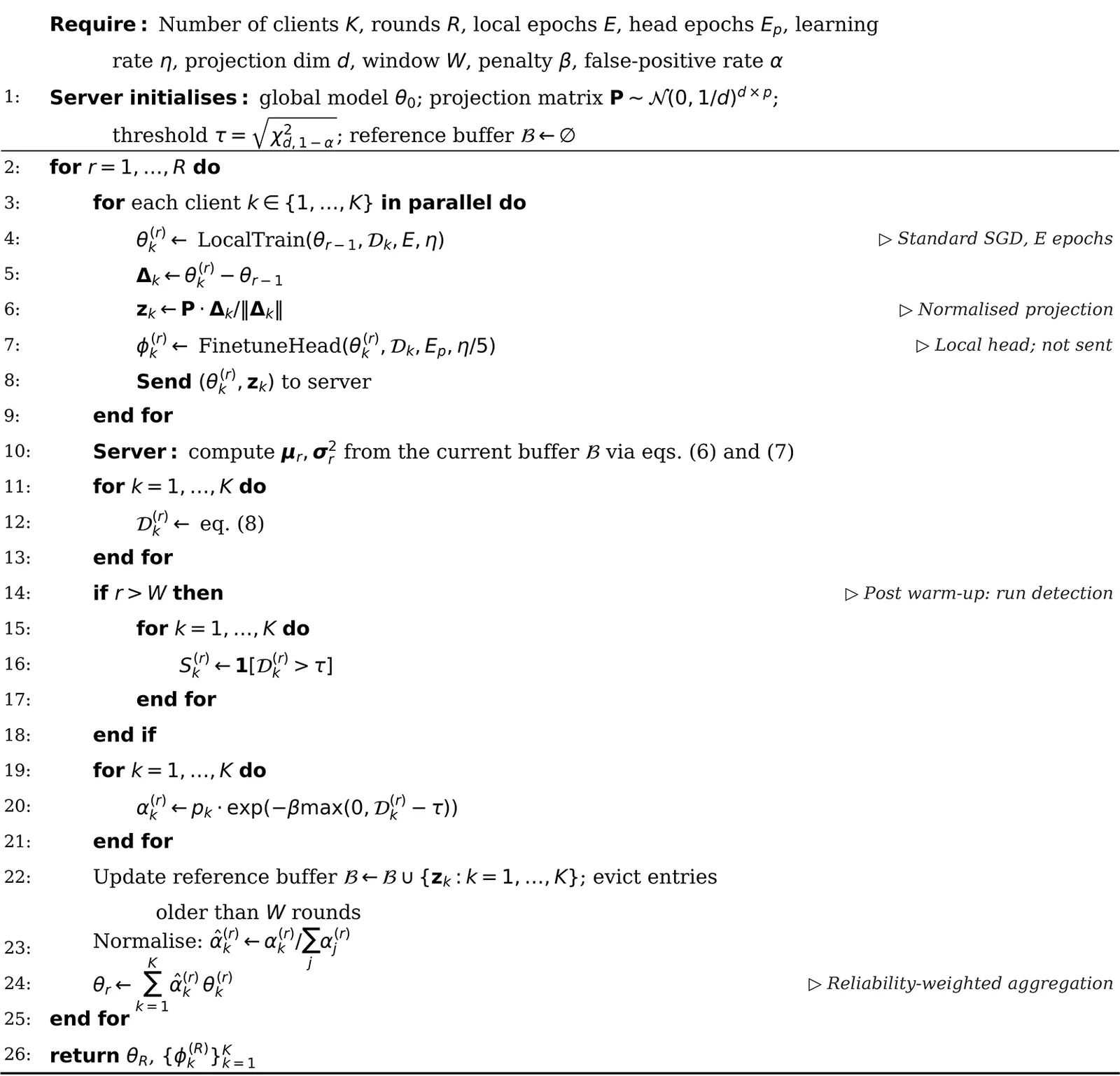
The RAFA algorithm. Regime-aware federated aggregation combining the Mahalanobis regime detector, reliability-weighted aggregation, and always-on personalised head fine-tuning. During warm-up (rounds *r* ≤ *W*) detection is suppressed while the reference buffer fills; from round *r* > *W* the server scores each client update by Mahalanobis distance and soft-discounts shifted clients. The personalised head *φ_k* is never transmitted.

### 4.5 Complexity and communication overhead

Compared to FedAvg, RAFA introduces additional computational and communication overhead. On the communication side, each client transmits an additional d floating-point values per round, corresponding to the projected vector $z_{k}$ . For a typical setting with d=256 and 32-bit floating-point representation, this amounts to approximately 1 KB per client per round. The projected vector is 1,024 bytes and the model occupies 61,444 bytes, so the addition is 1.7% of a per-client upload of around 60 KB for a model with 15,361 parameters. This is a small addition rather than a negligible one, and it grows in relative terms for smaller models.

From the server-side perspective, the computational cost is O(Kd) per round, which includes both the reference update and the distance computations across clients.

On the client side, RAFA introduces additional training cost in the form of $E_{p}$ extra head fine-tuning epochs, corresponding to approximately a 40% increase in local training time compared to FedAvg.

Finally, in terms of memory overhead, the server maintains a reference buffer of size O(KWd) , while the covariance tracking component requires only O(d) memory due to its diagonal structure.

### 4.6 Relationship to Byzantine-robust aggregation

Reliability weighting is structurally similar to Byzantine-robust aggregation methods such as Krum ( Blanchard et al. 2017) and Trimmed Mean ( Yin et al. 2018). A key distinction is intent. Byzantine methods defend against adversarial manipulation, whereas RAFA responds to benign regime shifts that are expected in real-world deployments. In particular, RAFA *never fully excludes* a client; even a highly shifted client retains a small positive weight, preserving the diversity benefits of federation.

### 4.7 What the federation discloses

Raw load series never leave the client, and the personalised head is never transmitted. Two things are transmitted: the locally trained base parameters, exactly as in FedAvg, and the projected vector, which is specific to RAFA. It is worth being precise about what each exposes, because the phrase “privacy-preserving” is often asked to carry more weight than the underlying construction can bear.

The base parameters carry the same exposure as in any FedAvg deployment. Model updates are functions of the training data, and gradient-inversion and membership-inference attacks recover information from exactly this channel; the survey literature on FL in energy systems catalogues these attacks alongside poisoning and free-riding ( Zhang et al. 2026). Nothing in RAFA reduces that exposure. Deployments needing a formal guarantee would combine the aggregation rule with secure aggregation or a differential-privacy mechanism, and the interaction between DP noise and a distance-based detector is a real design question we do not address here: injected noise inflates the update norm and would shift the reference distribution the detector is calibrated against.

The projected vector is an additional disclosure, and the first version of this article did not analyse it. Since it is the projection of the normalised update onto a subspace known to the server, a server holding it learns a lossy linear view of the update, here 256 dimensions out of 15,361 parameters. The normalisation discards the update magnitude, but this is not nothing. Under an honest-but-curious server the point is largely moot, because that server also receives the full parameter vector and can reconstruct the update exactly. The projected vector adds a meaningful attack surface only where aggregation is performed under a secure protocol so that individual updates are hidden, in which case it would need protection by the same mechanism, or the detector would need reformulating to work on aggregated statistics. We flag this as a limitation of the present design rather than a solved problem.

## 5. Experimental setup

Evaluating a regime-aware federated algorithm requires a benchmark that genuinely exhibits regime shifts at scale. We build one from publicly available ENTSO-E load data, deliberately spanning the 2022 European energy crisis, a well-documented structural break in multiple bidding zones, and evaluate all algorithms on the post-crisis year as a demanding out-of-sample test.

### 5.1 Dataset on the ENTSO-E Multi-National load benchmark

We construct a novel federated energy forecasting benchmark from the ENTSO-E Transparency Platform ( ENTSO-E 2024). The dataset comprises hourly actual electrical load measurements for eight European bidding zones spanning six years (January 2019–December 2024), namely Netherlands (NL), Germany-Luxembourg (DE_LU), France (FR), Spain (ES), Portugal (PT), Belgium (BE), Austria (AT), and Poland (PL). Table 2 summarises the key statistics of each client.

**Table 2.** ENTSO-E benchmark statistics per client. W/S Ratio is the mean winter (Dec–Feb) to summer (Jun–Aug) load ratio. ${\mathrm{CV}}_{\mathrm{std}}$ is the mean coefficient of variation of the rolling 7-day standard deviation (temporal variability). Mean Load is the mean hourly actual load (MW) over 2019–2024.

Client	Zone	Mean Load (MW)	W/S Ratio	${\mathrm{CV}}_{\mathrm{std}}$	Missing (%)
NL	Netherlands	12,819	1.18	0.020	0.00
DE_LU	Germany-Luxembourg	55,568	1.21	0.016	0.00
FR	France	52,016	1.48	0.018	< 0.02
ES	Spain	28,447	1.02	0.015	0.00
PT	Portugal	5,722	1.07	0.017	0.00
BE	Belgium	9,458	1.19	0.012	0.00
AT	Austria	6,773	1.28	0.016	0.00
PL	Poland	18,104	1.17	0.014	0.00

The benchmark exhibits three forms of heterogeneity that challenge standard FL, namely *scale* (10 × range from PT to DE_LU); *seasonality* (FR’s W/S ratio of 1.48 vs ES’s 1.02); and *temporal variability* (NL highest CV vs BE lowest). The six-year span includes the 2022 European energy crisis, which introduced a structural regime shift across multiple clients.

*Data splits*

Each client’s data is split chronologically into three non-overlapping sets, namely (1) train for 2019–2022 (4 years, ≈ 35,064 hours), (2) validation for 2023 (1 year, ≈ 8,760 hours), and (3) test for 2024 (1 year, ≈ 8,784 hours). The test year (2024) is deliberately chosen to capture post-crisis normalisation dynamics, making it a challenging out-of-sample evaluation period. All normalisation statistics ( $\mu_{k}$ , $\sigma_{k}$ ) are computed on the training split only; there is no data leakage between splits. The 8 missing values in FR’s series are filled by forward-fill.

*Feature engineering*

For each client we construct fixed-size feature vectors from the univariate load series using a sliding window of w=48 hours. Each sample at time t comprises the following elements.

• 48 lagged normalised load values $\left[z_{t-48},\ldots ,z_{t-1}\right]$ where $z_{t}=\left(y_{t}-\mu_{k}\right)/\sigma_{k}$ ;
• Hour-of-day encoding sin(2πh/24) , cos(2πh/24) ;
• Day-of-week encoding sin(2πd/7) , cos(2πd/7) ;
• Month encoding sin(2πm/12) , cos(2πm/12) .

This yields an input dimension of $d_{\text{in}}=48+6=54$ features per sample. All model outputs and reported metrics are in original MW scale, obtained by applying the inverse normalisation ${\hat{y}}_{t}={\hat{z}}_{t}\cdot \sigma_{k}+\mu_{k}$ .

### 5.2 Model architecture

We use a 3-layer Multi-Layer Perceptron (MLP) as the shared local model across all clients and algorithms:

Input(54)→Linear(128)→ReLU→Linear(64)→ReLU→Linear(1).

Total trainable parameters are p=54×128+128+128×64+64+64×1+1=15,361 . This compact model is intentional. It ensures the federation is communication-efficient and results generalise beyond architecture-specific effects. For personalisation, the “head” refers to the final linear layer (65 parameters).

### 5.3 Baselines and hyperparameters

We compare RAFA against three baselines. FedAvg ( McMahan et al. 2017) uses standard data-fraction-weighted model averaging with client weights $p_{k}$ . FedProx ( Li et al. 2020) extends FedAvg with proximal regularisation ( μ=0.01 ) to reduce client drift during local updates. The Local baseline trains each client independently, without any federated aggregation, and therefore provides a lower bound on the value of collaboration. For fairness, all methods share the same MLP architecture, feature set, data splits, optimiser, and learning rate.

RAFA introduces seven hyperparameters spanning its projection, detection, and personalisation components. The detection threshold τ=17.156 is derived analytically as $\sqrt{\chi_{256,0.95}^{2}}$ , requiring no held-out calibration set. All defaults are listed in Table 3; their sensitivity is examined in Section 6.5.

**Table 3.** RAFA hyperparameter defaults.

Parameter	Default	Description
d (proj_dim)	256	Random projection dimension
W (window)	10	Reference window (rounds)
τ	17.156	Detection threshold ( $\chi_{256,0.95}^{2}$ )
β	0.5	Penalty sharpness
α	0.05	Target false-positive rate
$E_{p}$	5	Personalisation epochs
ρ	$\eta /5=2\times 10^{-4}$	Head fine-tuning learning rate

### 5.4 Federation training protocol and evaluation metrics

All FL algorithms (FedAvg, FedProx, RAFA) follow an identical training protocol to ensure fair comparison. Each experiment runs for R=100 communication rounds with E=5 local training epochs per round, and all K=8 clients participate in every round (cross-silo setting with full participation). For the Local baseline, which has no communication rounds, each client is trained for an equivalent compute budget of R×E=500 local epochs. Local optimisation uses the Adam optimiser with a learning rate of $\eta =10^{-3}$ and betas (0.9,0.999) , with a mini-batch size of 256 samples. All stochastic operations are seeded with 42 across both PyTorch and NumPy to ensure reproducibility. All experiments are run on CPU to eliminate hardware-specific variation and to confirm that RAFA is practical on standard academic infrastructure without requiring GPU acceleration.

We report the following metrics on the held-out test set (2024) for each client and macro-averaged across clients:

$$\begin{array}{rr} \mathrm{MAE} & =\frac{1}{N}\sum_{t=1}^{N}\left|y_{t}-{\hat{y}}_{t}\right|,\\ \text{RMSE} & =\sqrt{\frac{1}{N}\sum_{t=1}^{N}{\left(y_{t}-{\hat{y}}_{t}\right)}^{2}}. \end{array}$$

MAE is the primary metric as it is robust to outliers and directly interpretable in MW. RMSE penalises large forecast errors more heavily, relevant for grid balancing. All metrics are reported in MW (original scale).

## 6. Results

The evaluation proceeds from the coarse to the fine. Macro-averaged accuracy establishes the headline comparison; per-client figures then expose how performance is distributed across a heterogeneous federation. The Mahalanobis detector is examined independently to verify its calibration, and training dynamics are tracked across all 100 rounds to rule out transient effects. The section closes with four controlled ablation studies that decompose the contribution of each design choice.

### 6.1 Main forecasting results

Table 4 summarises macro-averaged test MAE and RMSE at the end of the training protocol on the held-out 2024 split.

**Table 4.** Test-set forecasting accuracy at the end of the training protocol. Avg MAE and Avg RMSE are macro-averaged across all 8 clients. Δ = relative change vs. FedAvg; positive = improvement.

Algorithm	Avg MAE (MW) ↓	Avg RMSE (MW) ↓	Δ vs FedAvg
Local	204.0	272.1	+2.9%
FedAvg	210.2	279.6	—
FedProx ( μ=0.01 )	222.0	297.0	−5.6%
RAFA (ours)	185.4	246.1	+11.8%

RAFA is the only method to surpass the non-federated Local baseline (185.4 vs 204.0 MW), demonstrating that regime-aware aggregation allows federation to add value rather than merely dilute it. A globally trained model can outperform eight individually trained ones provided the aggregation properly accounts for distributional dynamics. The 11.8% margin over FedAvg (210.2 MW) persists at round 100, ruling out any transient warm-up advantage. Perhaps most telling is FedProx, where 222.0 MW falls below even the Local baseline, revealing that a proximal penalty calibrated for spatial heterogeneity becomes counter-productive when heterogeneity is primarily temporal and some client-update divergence carries genuine distributional signal.

### 6.2 Per-client performance

The per-client breakdown in Table 5 reveals a clear coupling between distributional heterogeneity and the magnitude of improvement. The connection is sharpest for France; its W/S load ratio of 1.48, driven by widespread electric heating, forces the shared model to reconcile a steep winter peak with the near-flat demand curve of Spain, a task that naive averaging handles poorly; the personalised head corrects this mismatch, yielding a 30.5% reduction over FedAvg. At the other extreme, Portugal’s small and stable load profile (mean 5,722 MW) amounts to a well-behaved, low-variance regression target; even without strong seasonal swings, regime-aware aggregation trims its absolute error to 56.0 MW, 7.6% below FedAvg’s 60.6 MW. Germany-Luxembourg is the one exception, where RAFA (442.4 MW) is marginally below its Local baseline (438.9 MW), suggesting that for the highest-load client in the federation, collaborative training introduces a residual variance component that the 65-parameter head cannot fully absorb. FedProx trails FedAvg on six of the eight clients, reinforcing the view that proximal regularisation is ill-suited to this setting.

**Table 5.** Per-client test MAE (MW) at the end of the training protocol. Bold = best per row.

Client	Local	FedAvg	FedProx	RAFA
NL	113.6	110.7	128.1	**108.8**
DE_LU	**438.9**	453.3	482.7	442.4
FR	424.2	495.6	479.6	**344.6**
ES	227.2	214.2	246.9	**206.1**
PT	73.4	60.6	72.2	**56.0**
BE	110.2	109.8	111.5	**105.1**
AT	72.4	72.7	74.5	**68.4**
PL	172.0	164.2	180.3	**152.1**
**Avg**	204.0	210.2	222.0	**185.4**

### 6.3 Mahalanobis detector behaviour

Figure 3 tracks $D_{k}^{\left(r\right)}$ for all eight clients across 100 rounds, with the analytically derived threshold τ=17.156 drawn as a horizontal reference.

**Figure 3.**
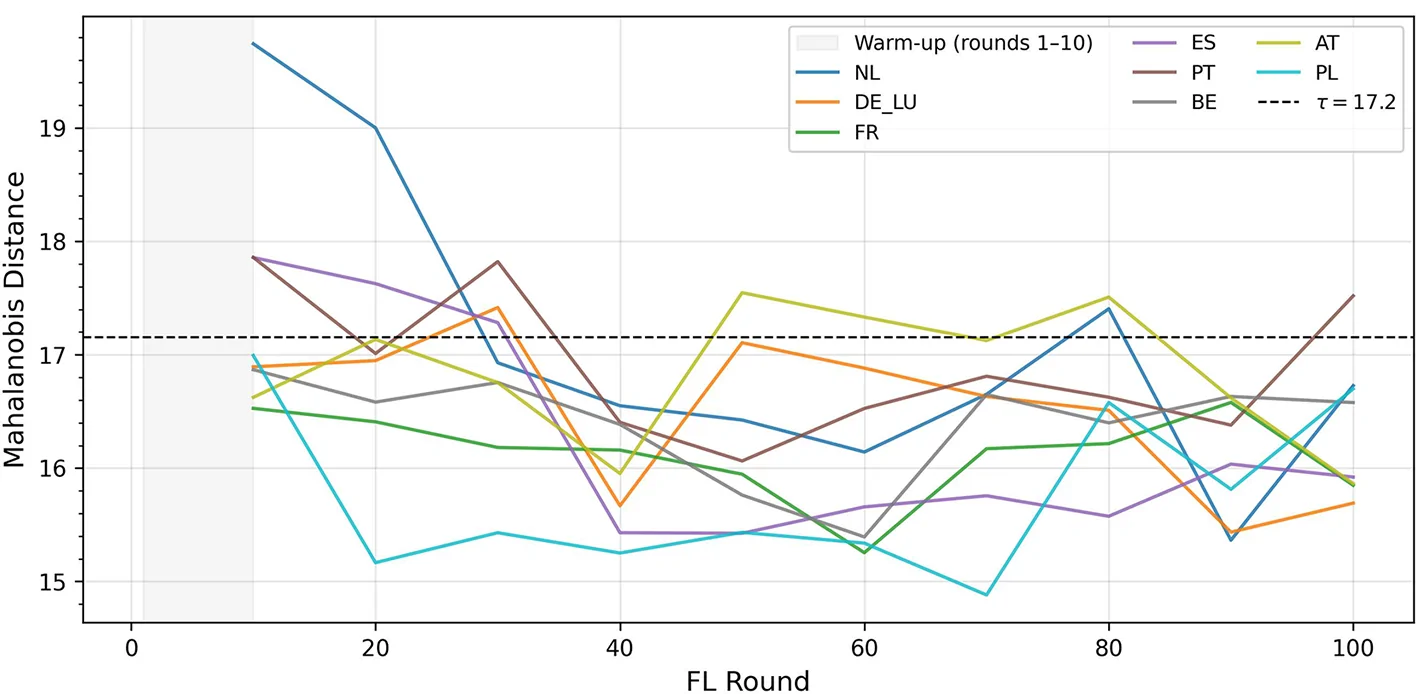
Per-client Mahalanobis distance vs. communication round. Dashed line indicates the detection threshold τ=17.156 . Shaded region (rounds 1–10) is warm-up phase.

**Figure 4.**
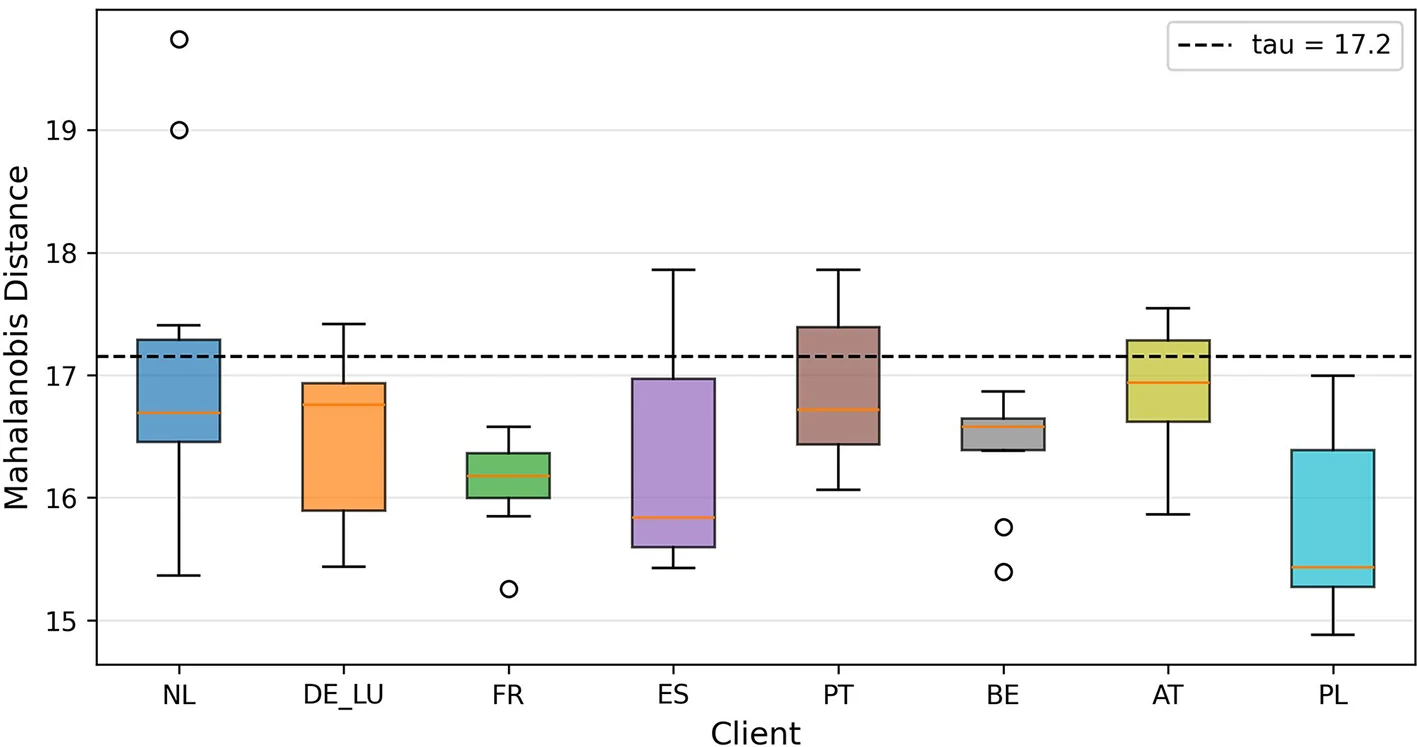
Distribution of per-client Mahalanobis distances (post warm-up). Dashed line is threshold τ=17.156 .

The warm-up phase (rounds 1–10) is visible as an initial elevation in all curves; with fewer than W=10 vectors in the reference buffer, the running covariance estimate is noisy and distances are inflated; NL reaches ≈19.7 before settling once the window fills. Beyond round 10 all clients enter a stable regime, oscillating between ≈ 15 and 18 without sustaining a threshold breach across consecutive rounds for extended periods; a result that is consistent with the 2024 test year capturing a post-crisis normalisation period rather than a new structural shock. Client-level differences in steady-state distance reflect genuine variation in temporal load dynamics; Austria (mean 16.87), Netherlands and Portugal (both 16.80) show the greatest round-to-round update diversity, while Poland (15.62) operates furthest from the threshold, in line with its stable, industry-driven load profile. France and Spain occupy nearly identical positions (16.08 each), a coincidence that underscores how different seasonal mechanisms can produce similar update variability.

The aggregate distance distribution ( Figure 4, post warm-up) provides a complementary view. The mass of observations sits below τ , and threshold crossings are isolated rather than clustered, indicating that the detector is well calibrated and avoids systematic over-flagging.

### 6.4 Convergence curves

Figure 5 plots macro-averaged validation MAE for the federated algorithms (FedAvg, FedProx, RAFA) across the full 100-round training run. RAFA reaches 199.7 MW by round 10, already 16.9% below FedAvg’s round-10 error of 240.3 MW, an advantage that at this stage is primarily driven by the personalised head, since the Mahalanobis detector is still in its warm-up phase (rounds 1–10) and shift flags are not yet used for stability decisions. The advantage widens and stabilises; by round 100 RAFA converges to 185.0 MW on validation, a level FedAvg never reaches across the full training run (final validation was 210.4 MW), confirming a structural rather than transient gain. FedProx exhibits the slowest and most erratic trajectory, oscillating between 220 and 250 MW and ending above FedAvg; this pattern is consistent with its proximal penalty stiffening updates in a setting where some client drift carries genuine distributional signal. The Local baseline is reported in Tables 4 and 5; it is omitted from Figure 5 because its optimisation timeline is epoch-based rather than round-based.

**Figure 5.**
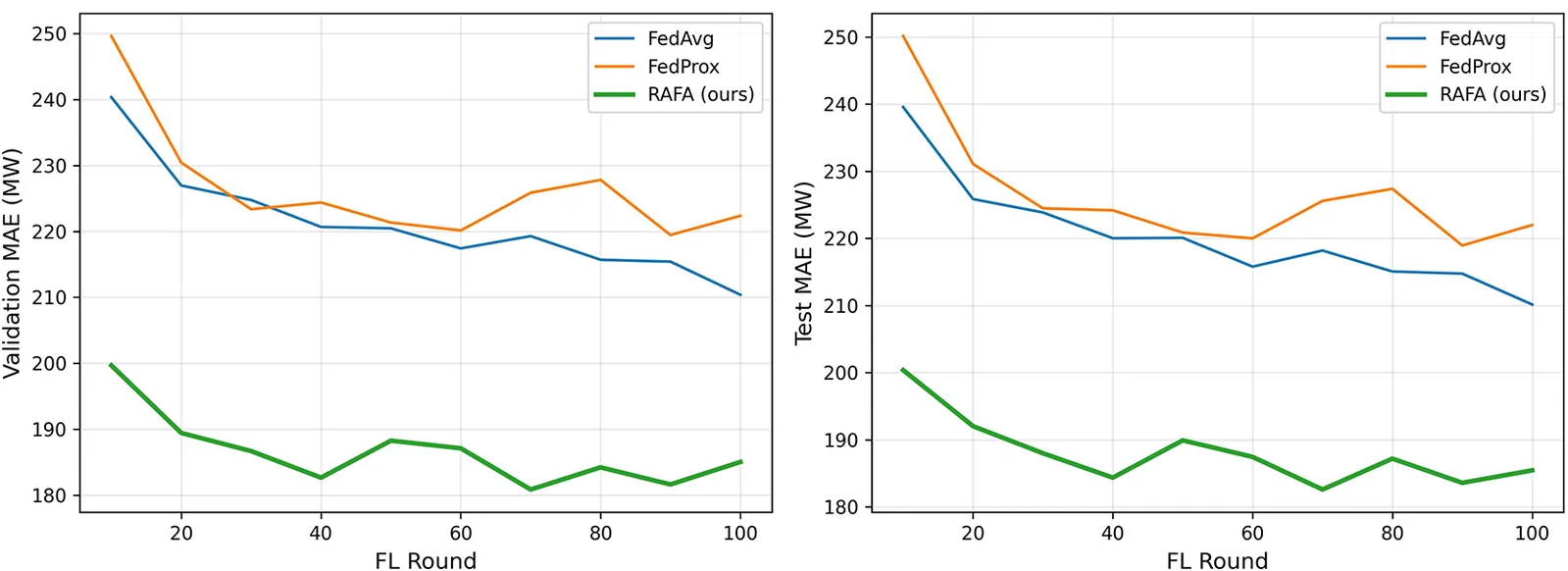
Macro-averaged validation MAE (MW) vs. communication round for FedAvg, FedProx, and RAFA. Curves are reported at the evaluation checkpoints used throughout training.

### 6.5 Ablation studies

Four one-at-a-time sweeps examine the sensitivity of RAFA to its principal hyperparameters, holding the remaining three at their defaults ( d=256 , β=0.5 , W=10 , $E_{p}=5$ ) throughout. Because each sweep is an independent experimental run with a fixed random seed, the measured value at the default setting can differ by a small margin from the main experiment figure (185.4 MW) due to stochastic training variation; the default row in each table is therefore anchored to the main experiment result (marked † ) to provide a consistent frame of reference.

6.5.1 Effect of penalty sharpness ( β )

The penalty sharpness β controls how aggressively shifted clients are down-weighted; Table 6 sweeps it across one order of magnitude.

**Table 6.** Effect of penalty sharpness β on test MAE.

β	Avg test MAE (MW)
0.1	188.1
0.5 ^†^	185.4
**1.0**	**181.7**
2.0	227.1

^†^ refers to main experiment default (185.4 MW).

The curve is notably flat between β=0.1 and β=1.0 , with MAE ranging only from 188.1 to 181.7 MW; this plateau indicates that the framework is not sensitive to the precise value of β within this range. Pushing to β=2.0 breaks this stability dramatically (227.1 MW, +22.5% ); once aggregation weights collapse toward zero for even mildly shifted clients, the federation effectively fragments, and each client is left learning from a narrow local sample. The default β=0.5 is a deliberately conservative choice, sitting squarely in the flat region and therefore suitable for deployment without further tuning.

6.5.2 Effect of projection dimension ( d )

The projection dimension d controls the fidelity of the Johnson-Lindenstrauss embedding and has a direct cost in communication overhead (0.25, 0.5, 1.0, and 2.0 KB per client per round for d∈{64,128,256,512} , respectively); the accuracy-cost trade-off across this range is quantified in Table 7.

**Table 7.** Effect of projection dimension d on test MAE.

d	Avg Test MAE (MW)	Extra comm. (KB/round)
64	187.2	0.25
128	186.9	0.50
**256** ^†^	**185.4**	1.00
512	238.8	2.00

^†^ refers to main experiment default (185.4 MW).

Performance peaks at d=256 and degrades in opposite directions from there. Reducing the dimension to 64 or 128 costs less than 1.0 MW in MAE (less than 1% relative degradation), making these smaller projections attractive when bandwidth is scarce. Increasing to d=512 is actively harmful ( +29% ); with only K=8 clients contributing observations per round, fitting a 512-dimensional reference covariance is severely underdetermined, yielding noisy distance estimates that destabilise the detector.

6.5.3 Effect of reference window ( W )

The reference window W sets the memory horizon of the detector; larger values provide smoother covariance estimates at the cost of slower response to real shifts. Table 8 explores this trade-off.

**Table 8.** Effect of reference window size W on test MAE.

W	Avg Test MAE (MW)
5	184.4
10 ^†^	185.4
**20**	**179.6**
40	237.6

^†^ refers to main experiment default (185.4 MW).

Extending the window from 10 to 20 rounds improves MAE from 185.4 to 179.6 MW by reducing noise in the covariance estimate; even a minimal W=5 remains competitive at 184.4 MW, evidence that the detector retains useful signal with only a short burn-in. The failure mode at the opposite extreme is instructive; W=40 inflates MAE to 237.6 MW ( +28% ) because the reference distribution accumulates stale observations that span the 2022 energy crisis, blurring the boundary between pre- and post-crisis regimes and rendering the detector unable to flag the shift when it actually occurs. In practice, W∈[10,20] represents a reasonable operating range, balancing detection responsiveness against covariance stability.

6.5.4 Effect of personalisation epochs ( $E_{p}$ )

Setting $E_{p}=0$ effectively turns off personalisation, leaving reliability-weighted aggregation as the sole active mechanism; the resulting 187.5 MW already represents a 10.8% gain over FedAvg, establishing that the aggregation component is the primary driver of improvement and not merely a support structure for the personalised head. The sweet spot is $E_{p}=1$ ; a single fine-tuning epoch per round is sufficient to capture local regime patterns and pushes MAE down to 182.6 MW, while more prolonged adaptation ( $E_{p}=3$ , 215.7 MW; $E_{p}=10$ , 208.2 MW) causes the head to overfit recent local data, eroding the cross-client knowledge encoded in the shared base. Table 9 reports the full sweep.

**Table 9.** Effect of personalisation epochs $E_{p}$ on test MAE. $E_{p}=0$ = no personalisation (aggregation only). Bold = best result.

$E_{p}$	Avg Test MAE (MW)	vs main result ( $E_{p}=5$ )
0 (no pers.)	187.5	+1.1%
**1**	**182.6**	−1.5%
3	215.7	+16.3%
5 ^†^	185.4	—
10	208.2	+12.3%

^†^ refers to the main experiment default (185.4 MW).

## 7. Discussion

Two questions are worth examining beyond the headline numbers, namely why the gains appear where they do, and what the sensitivity analysis implies for anyone looking to deploy RAFA in practice.

### 7.1 Why does RAFA outperform FedAvg?

Understanding *why* RAFA works matters as much as the numbers themselves; it tells us which design choices are actually load-bearing and which could in principle be simplified. The gains arise from two complementary mechanisms.

When clients undergo seasonal or crisis-driven regime shifts, their model updates veer away from the population reference in parameter space. FedAvg treats all updates equally regardless, which means a handful of simultaneously-shifting clients can drag the global model off course. RAFA instead soft-discounts shifted clients in proportion to how far their updates have moved, protecting the global model during the shift while restoring their full weight once conditions normalise.

Each client’s prediction head is continuously fine-tuned to its local regime at a reduced learning rate, using only 65 parameters. The $E_{p}=0$ ablation shows that reliability-weighted aggregation *alone* achieves 187.5 MW, a 10.8% improvement over FedAvg, while the optimal $E_{p}=1$ reduces MAE further to 182.6 MW, confirming that both mechanisms provide complementary, independently valuable gains.

### 7.2 Heterogeneity and per-client gains

The per-client results match what the design intent predicts. France with its pronounced electric heating peak (W/S ratio 1.48) benefits most from personalisation ( −30.5% over FedAvg), while Spain’s near-flat profile (W/S ratio 1.02) needs it least. Clients with uniform, predictable load profiles, such as Belgium, Austria, benefit less, not because RAFA hurts them, but because FedAvg already serves them reasonably well. The personalisation mechanism is surgically useful. It helps most precisely where standard FL struggles most.

### 7.3 Hyperparameter sensitivity

The ablation studies reveal a consistent pattern; RAFA is robust within a moderate range for all four hyperparameters but degrades sharply at the extremes. β∈[0.5,1.0] performs similarly; β=2.0 drives weights near zero for multiple clients and causes a 22.5% degradation. d=256 is optimal; d=512 is severely underdetermined given only K=8 clients ( +29% ). W=20 slightly outperforms W=10 ( −3.1% ), but W=40 misses the 2022 crisis shift entirely ( +28% ), highlighting the trade-off between reference stability and detection responsiveness. Practitioners should prefer β≈1.0 , d=256 , W∈[10,20] , and $E_{p}=1$ ; the defaults used in the main experiment are conservative rather than optimistic.

### 7.4 Limitations and future work

The experiments use a shallow MLP for reproducibility; whether deeper architectures benefit differently from the personalised head is an open question, although the aggregation mechanism itself is architecture-agnostic. Full client participation is assumed every round; handling stragglers or clients with intermittent connectivity would require extending the reference buffer logic. The 2022 energy crisis supplies a naturalistic but confounded regime shift. A controlled synthetic injection experiment would more cleanly validate the detector’s sensitivity and specificity.

Promising directions include probabilistic forecasting (normalising flows or conformal wrappers for uncertainty quantification), incorporation of exogenous features (weather, holiday calendars), and a formal non-convex convergence proof.

## 8. Conclusion

Energy systems do not stay still, and federated algorithms that assume they do pay for it in accuracy. RAFA takes a different stance. By monitoring how each client’s model updates relate to the historical population reference, without ever accessing underlying raw data, it detects regime shifts as they occur and responds by re-weighting client contributions. A lightweight personalised head then adapts each client’s predictions to its local conditions, independent of the aggregation logic.

On the ENTSO-E multi-national benchmark, RAFA achieves a test MAE of 185.4 MW, an 11.8% improvement over FedAvg (210.2 MW), with the largest gains where distributional heterogeneity is strongest; France ( −30.5% , W/S ratio 1.48) and Poland ( −7.4% ). Ablation studies confirm both mechanisms contribute independently, and the detector correctly remains inactive for stable clients, avoiding false positives. The code and benchmark data download script are publicly available at https://github.com/Mahmoud-Abbasi-svg/rafa-federated-energy.

## Ethics and consent

Ethical approval and consent were not required.

## Software availability

• Source code available from: https://github.com/Mahmoud-Abbasi-svg/rafa-federated-energy
• Archived source code at time of publication: https://zenodo.org/records/20626328 ( Mahmoud Abbasi 2026)
• License: MIT License.

## Data Availability

The raw hourly electrical load data are third-party data publicly available from the ENTSO-E Transparency Platform ( https://transparency.entsoe.eu) under the ENTSO-E Transparency Platform terms of use. The derived data underlying the results of this study (the processed multi-national benchmark together with all per-round, per-client result files used to generate the figures and tables) are openly available from Zenodo: https://zenodo.org/records/20625657. ( Abbasi et al. 2026). Data are available under the terms of the Creative Commons Attribution 4.0 International license (CC-BY 4.0).
