# Supplementary material for: Regime-Aware Federated Aggregation (RAFA) for privacy-preserving energy load forecasting across heterogeneous European grid clients
Source: Open Res Eur. 2026 Aug 6;6:228. Originally published 2026 Jul 3. [Version 2] doi: 10.12688/openreseurope.24355.2 (PMC13627948; doi:10.12688/openreseurope.24355.2)
Supplement: Peer review history

# Reviewer report: Reviewer response for version 2

**DOI:** 10.21956/openreseurope.26842.r80193

**Published:** 2026-09-30

**Authors:** Sandhya Patidar

**Affiliations:** 1 Heriot-Watt University, Edinburgh, UK

**Importance and research need**: This paper proposes RAFA (Regime-Aware Federated Aggregation), a federated learning (FL) framework designed for short-term electricity load forecasting under non-stationary conditions. Federated learning is becoming increasingly relevant to several applications in smart energy systems, including smart grids, smart meters, energy communities, cross-border electricity forecasting, and privacy-aware AI. The paper therefore addresses an important and timely research problem.

 Standard FL methods such as FedAvg are not explicitly designed to account for temporally evolving client distributions, whereas electricity demand evolves due to seasonality, behavioural changes and any major/rare events such as the 2022 European energy crisis.  Much existing FL research focuses on static non-IID data rather than temporal non-stationarity. In this context, the paper addresses this gap by proposing RAFA, which aims to detect regime shifts from model updates rather than requiring access to clients' raw data.

**Methodology and novelty**: The paper develops a methodological framework combining three main components: Mahalanobis-distance-based regime detection, reliability-weighted federated aggregation, and personalised local prediction heads. The methodology is well organised, with clear mathematical notation and an easy-to-follow workflow. The algorithm incorporates random projection, a Mahalanobis detector, adaptive aggregation weighting, and personalised prediction heads, and is supported by pseudocode and an adequate discussion of computational complexity.

 None of these individual components appears fundamentally novel in isolation. The paper itself appropriately acknowledges that Mahalanobis-based out-of-distribution detection is established, FedPer already employs local personalisation, reliability weighting has similarities with robust/Byzantine aggregation approaches, and random projection is well known. Thus, the contribution is primarily integrative, combining these ideas within a regime-aware federated framework for energy-load forecasting, rather than introducing an entirely new underlying algorithmic principle.

 This represents a useful methodological and engineering contribution. However, the extent to which the regime-aware aggregation component itself provides a distinct methodological advance requires careful consideration in light of the revised experimental results.

**Experimental design: ** The experimental design is one of the paper's strengths. It uses six years of hourly electricity-load data across eight European countries representing heterogeneous demand and climatic conditions, including the period encompassing the 2022 European energy crisis. This provides a useful and challenging real-world setting for evaluating federated forecasting across geographically heterogeneous clients. However, as discussed below, data spanning multiple historical regimes should be distinguished from exposing the FL system to dynamically evolving client distributions during training.

**Analysis: **Version 2 reports results over multiple random seeds and standard deviations. Nevertheless, inferential support for comparisons between methods could be strengthened. Any statistical significance analysis should account for the paired and temporally dependent nature of forecasting errors. Rather than treating individual hourly errors as independent observations, the authors could consider suitable block-based resampling or other paired procedures that preserve temporal dependence. Reporting confidence intervals for differences in predictive performance between competing methods would also help establish whether the observed improvements are robust.

**Results**: Based on the available information, the original version appears to report substantial improvements for RAFA relative to FedAvg, with improvements of approximately 12% overall and, for some individual clients such as France, more than 30%. Version 2, however, substantially changes how these improvements are interpreted. Following revisions to the experimental controls and workflow, the detector appears to activate infrequently on the principal benchmark. The authors acknowledge that when no client exceeds the detection threshold, the reliability weights reduce to the standard aggregation weights, and the resulting aggregation becomes mathematically equivalent to FedAvg. This finding weakens the evidence that regime-aware aggregation is the principal mechanism responsible for RAFA's reported predictive gains. The revised analysis instead indicates that much of the improvement may be attributable to personalised local adaptation.

 Nevertheless, RAFA remains a useful engineering contribution and provides a potentially valuable integration of several existing techniques. The issue is therefore not that the framework lacks value, but that the empirical evidence in Version 2 appears to support a more incremental interpretation of the regime-aware aggregation contribution than some of the manuscript's original claims suggest. The claims about the source of RAFA's performance gains should therefore align more closely with the revised evidence. In particular, the paper should establish more clearly under what conditions regime-aware weighting becomes active and provides an advantage beyond an otherwise equivalent personalised federated model.

**Discussion: **The discussion is generally balanced, and the authors acknowledge several important limitations, including uncertainty estimation and the need to evaluate the framework under controlled distribution shifts. The discussion could nevertheless more clearly distinguish the three related but different phenomena of cross-client heterogeneity, update-space anomaly detection, and within-client temporal regime change. These distinctions are central to interpreting what the proposed detector is actually identifying.

**Open science practice:** The paper provided source code, links to the Zenodo archive and GitHub repository, demonstrating good open science practice.

**Overall assessment**: Overall, the manuscript is well written and has appeared to be substantially improved relative to Version 1 following the reviewers' comments. The additional experiments and methodological clarifications requested should have improved its transparency and scientific value.

 Nevertheless, several scientific issues remain unresolved, particularly regarding the detector's interpretation, the relationship between regime detection and temporal non-stationarity, and the extent to which regime-aware aggregation explains the reported forecasting improvements. Addressing the following points would further strengthen the methodological rigour and clarify the proposed framework's contribution.

1. The authors explicitly acknowledge that the chi-square calibration is approximate and that it considers an empirically calibrated threshold. However, the default detector still relies on strong assumptions regarding the approximate Gaussian of projected updates, the use of diagonal covariance, and the suitability of the chosen reference distribution. With relatively few reference observations, uncertainty in estimating the corresponding distributional properties may remain substantial. I therefore recommend that the authors more clearly distinguish the chi-square threshold as a heuristic or approximate operating threshold rather than a formally calibrated significance threshold. It would also be useful to compare the analytical and empirical calibration procedures in terms of false-positive rates, detection power, and detection latency under controlled shifts. More flexible adaptive or non-parametric calibration strategies could also be considered as future extensions [ REF].
2. The per-client reference-buffer ablation is useful and helps address an important conceptual issue. The default detector nevertheless compares a client's update against a pooled reference distribution. As the authors recognise, pooled referencing can potentially combine two different effects: within-client temporal change + persistent cross-client heterogeneity. Consequently, a large pooled Mahalanobis distance could indicate either that a client's behaviour has changed over time or simply that the client remains systematically different from other members of the federation. This distinction is fundamental because a pooled reference primarily evaluates whether a client is atypical relative to the federation, whereas a temporal regime shift is more naturally interpreted as a deviation from that client's own historical behaviour. I therefore recommend that the authors expand the per-client historical reference analysis already introduced in Version 2, rather than treating it only as a secondary ablation. A direct comparison between pooled and client-specific reference schemes, including their respective detection rates, false positives, and forecasting consequences, would clarify whether the proposed detector primarily identifies persistent cross-client heterogeneity or genuine within-client temporal change [ REF, REF].
3. The train-validation-test split follows a logical chronological structure: the training split covers 2019–2022, validation uses 2023, and testing uses 2024. However, each FL communication round appears to perform local optimisation using the same underlying 2019–2022 training partition. Consequently, the client data-generating distribution does not explicitly evolve from one communication round to the next in the principal training experiment. Round-to-round changes in model-update vectors may therefore reflect optimisation dynamics, random mini-batch selection, and stochastic training variability rather than temporal concept or regime drift. This raises a fundamental question: to what extent does the main experiment demonstrate that RAFA detects evolving temporal regimes? Earlier reviewers also raised this issue. The controlled shift-injection experiment in Version 2 is useful because it allows evaluation of detector behaviour when a shift is deliberately introduced. However, this demonstrates detector performance under an imposed shift rather than establishing that the main European energy dataset experiment constitutes online regime detection. The manuscript should therefore distinguish clearly between: forecasting using data that contain heterogeneous historical regimes; detecting anomalous or atypical model updates; and detecting temporal regime changes as client data evolve during federated deployment. A genuinely sequential experiment, for example using chronologically arriving observations, expanding windows, or sliding-window training, would provide substantially stronger validation of the third claim [ REF].
4. The manuscript defines a regime shift in terms of KL divergence between latent client data distributions but operationally detects shifts through Mahalanobis distance in projected model-update space. The conceptual motivation is reasonable, but the relationship between these two quantities is not sufficiently established. More specifically, a change in *Pk(X,Y) *does not necessarily imply a detectable change in the direction or geometry of the normalised model update. Conversely, optimisation dynamics may produce substantial changes in update vectors even without a corresponding change in the underlying client data distribution. Thus, the theoretical definition and the operational Mahalanobis detector should not be treated as directly interchangeable without further justification. The authors should therefore frame Mahalanobis distance in projected update space more explicitly as a proxy or operational indicator of latent distributional change and discuss the conditions under which changes in the underlying client distribution are expected to manifest as detectable changes in the model-update representation. This would strengthen the conceptual connection between the formal definition of regime shift and the actual RAFA algorithm.
5. Check and update Table 9 (Section 6.5.4) and Section 7.1, reporting that an aggregation-only configuration (Ep=0) has MAE = 187.5 MW versus 210.2 MW for FedAvg, and interpret this as a 10.8% gain attributable to reliability-weighted aggregation. This contradicts the version 2 amendments that state that – “[ *On this benchmark the personalised head accounts for essentially all of the gain, and regime-aware aggregation contributes little, because no client exceeds the detection threshold after warm-up and the aggregation is then arithmetically identical to FedAvg.]*” The authors should clarify this discrepancy and verify that all comparisons use equivalent experimental conditions, including training budgets, random seeds, model initialisation, warm-up procedures, aggregation rules, and evaluation protocols. It would also be helpful to report detector activation frequencies and the actual deviation of RAFA aggregation weights from the corresponding FedAvg weights for the experiments underpinning Table 9.
6. The discussion of privacy appropriately acknowledges that keeping raw data local does not by itself provide a formal privacy guarantee. I recommend maintaining this distinction consistently throughout the title, abstract, plain-language summary and conclusions. In particular, "privacy-preserving" should either be carefully qualified as referring to raw-data locality or replaced by wording such as "privacy-aware" or "federated" unless a formal privacy mechanism or guarantee is evaluated. The interaction of RAFA with differential privacy and secure aggregation is an important direction because noise or hidden individual updates may directly affect the proposed distance-based detector.

Is the study design appropriate and does the work have academic merit?

Partly

Is the work clearly and accurately presented and does it cite the current literature?

Partly

If applicable, is the statistical analysis and its interpretation appropriate?

Partly

Are all the source data underlying the results available to ensure full reproducibility?

Yes

Are the conclusions drawn adequately supported by the results?

Partly

Are sufficient details of methods and analysis provided to allow replication by others?

Yes

Reviewer Expertise:

My research focuses on application of mathematical, statistical, data science and machine learning approaches for energy, climate, environmental and infrastructure systems. Key areas include explainable and trustworthy AI, uncertainty quantification, extreme value theory, time-series modelling, forecasting, clustering, anomaly detection, digital twins, smart energy systems, climate-risk analysis, hydrology, flood risk, water quality and resilient infrastructure decision support.

I confirm that I have read this submission and believe that I have an appropriate level of expertise to confirm that it is of an acceptable scientific standard, however I have significant reservations, as outlined above.

# Reviewer report: Reviewer response for version 1

**DOI:** 10.21956/openreseurope.26376.r77718

**Published:** 2026-07-25

**Authors:** Óscar García

**Affiliations:** 1 UNIR—International University of La Rioja, Logroño, Spain

The manuscript proposes RAFA, a federated learning framework combining a Mahalanobis-distance detector on random-projected client updates, reliability-weighted aggregation, and always-on personalised head fine-tuning, evaluated on a six-year ENTSO-E multi-national load benchmark covering eight bidding zones. The problem is well motivated, the method is clearly formulated, the related-work coverage is current, and the code and derived data are openly archived. My reservations concern internal numerical inconsistencies, an experimental protocol that does not appear to instantiate the temporal regime shifts the method targets, and the absence of variance estimates and of a key control baseline.

 The framework is coherent, the implementation is openly available, and the topic is relevant to European grid operators. Points 1, 2, 3, 5 and the parameter-count and overhead corrections must be addressed for the article to be scientifically sound; points 4, 6, 7 and 8 would substantially strengthen it. Most of these require re-running existing experiments with additional seeds and one extra baseline rather than a new experimental campaign.

 There are some  points that should be addressed:

- Warm-up behaviour. The text states that detection is suppressed during the warm-up rounds, but in the algorithm the reliability weights appear to be computed unconditionally at every round, precisely when the reported distances are most inflated; please reconcile the description with the implementation and clarify the role of the shift flag, which is computed but never subsequently used.
- Plausibility of the aggregation-only ablation. Given that the method is shown to reduce to exact FedAvg when no client exceeds the threshold, and that threshold crossings are reported as isolated and marginal, it is unclear how the aggregation mechanism alone can account for the improvement attributed to it, and the authors should report how often clients are actually flagged and how far the resulting aggregation weights depart from the standard data-fraction weights.
- Whether the benchmark exercises temporal shift. As described, every round trains on the same fixed training split, so client distributions do not evolve during federated training and the observed distances may reflect stochastic variability in updates rather than genuine regime change; the authors should either clarify that data are streamed across rounds or carry out the controlled shift-injection experiment currently deferred to future work, since several interpretations that link round indices to calendar events depend on this point.
- Missing control baseline. Because personalisation is applied unconditionally, a FedAvg variant with an identical personalised head is needed to isolate the contribution of regime-aware aggregation, and comparison against a centralised model and at least one drift-aware or personalised federated method would substantiate the positioning relative to the related work.
- Statistical support and hyperparameter selection. All results derive from a single seed with no dispersion measures, several ablation differences are small enough to fall within plausible run-to-run variation, and one sweep is non-monotonic in a way the stated explanation cannot account for; results should be averaged over multiple seeds with standard deviations, and hyperparameters should be selected on the validation split rather than on the test split.
- Calibration of the detection threshold. The chi-squared threshold assumes a known mean and covariance and approximately independent Gaussian components, whereas here the reference statistics are estimated from fewer observations than dimensions and the updates are normalised before projection, so the nominal false-positive rate is unlikely to hold and either a corrected or empirically calibrated threshold, or an explicit acknowledgement of the approximation, is warranted.
- Detector semantics. The reference distribution is pooled across all clients and rounds, so the score measures deviation from the cross-client population rather than from each client's own history, which conflates spatial heterogeneity with the temporal shift the paper sets out to detect; a per-client reference buffer would be a natural ablation, and the near-identical scores obtained for clients with opposite seasonal profiles should be discussed as a limitation of discriminative power rather than as a coincidence.
- Attribution and aggregation of the gains. The largest per-client improvement is attributed to personalisation although the corresponding ablation is only reported as a macro-average, so a per-client breakdown is needed; in addition, since client loads span an order of magnitude, a scale-free aggregate metric should accompany the macro-averaged MAE.
- Numerical and formulation details. Several derived quantities, equation renderings and figure captions do not verify against the values reported in the text, so a systematic check of all arithmetic, notation and cross-references throughout the manuscript is needed; as one example, the parameter count in Section 5.2 evaluates to 15,361 rather than the stated 15,489, which in turn affects the communication-overhead percentage given in Section 4.5. Particular attention should be paid to symbols used with two distinct meanings and to the unambiguous specification of whether the personalised head is included in θ, in Δ_k and in the projection, as this is essential for replication.
- References and citations. The reference list and its in-text usage require a careful revision for bibliographic accuracy, author-name parsing, consistency between text and list, and correspondence between each citation and the claim it supports; as one example, the FedProx entry (Li et al. 2020) appears under a title that does not match the published paper. I would also encourage replacing preprint entries with their peer-reviewed versions where available and softening attributions such as "definitive taxonomy", "first systematic survey" and "state of the art" unless directly supported by comparative evidence. Relatedly, the statement that privacy is "preserved by construction" is too strong given the gradient-inversion and membership-inference literature the manuscript itself cites, and the additional leakage surface of the transmitted projected vector is not analysed.

Finally, writing should be also reviewed due to typos and references.

Is the study design appropriate and does the work have academic merit?

Yes

Is the work clearly and accurately presented and does it cite the current literature?

Partly

If applicable, is the statistical analysis and its interpretation appropriate?

Yes

Are all the source data underlying the results available to ensure full reproducibility?

Yes

Are the conclusions drawn adequately supported by the results?

Yes

Are sufficient details of methods and analysis provided to allow replication by others?

Yes

Reviewer Expertise:

Energy, Big Data, Artificial Intelligence

I confirm that I have read this submission and believe that I have an appropriate level of expertise to confirm that it is of an acceptable scientific standard.

# Reviewer report: Reviewer response for version 1

**DOI:** 10.21956/openreseurope.26376.r76969

**Published:** 2026-07-23

**Authors:** Paula Lamo Anuarbe

**Affiliations:** 1 Universidad de Cantabria, Santander, Cantabria, Spain

I found this work to be an interesting and well-motivated manuscript addressing a relevant problem in federated learning for energy systems. The paper is generally well organised, the motivation is convincing, and the proposed methodology is presented in a clear and logical way. The experimental setting based on ENTSO-E data is also highly relevant for the European research community. My comments below are intended to help strengthen the presentation and improve the clarity of the contribution:

 A) The practical interpretation of the detected regime shifts could be explained a little further. While the mathematical formulation of the detector is very clear, it would be helpful to briefly discuss how the detected events may relate to real-world situations such as seasonal transitions, the 2022 European energy crisis, or other operational changes affecting electricity demand. This would make the contribution easier to appreciate for readers coming from the energy domain without requiring any additional experiments.

 B) The discussion on the selected hyperparameters could be slightly expanded. The default values are clearly presented, but a short comment indicating which parameters have the greatest practical influence, and which ones appear to be relatively robust would make the proposed method easier to adopt in future work.

 C) The originality of RAFA could be highlighted even more clearly by expanding the comparison with existing personalisation approaches such as FedPer or LG-FedAvg. A brief discussion explaining the conceptual differences would help readers identify the novelty of the proposed framework more immediately.

 D) The section discussing computational overhead is useful. Since the personalised head fine-tuning increases the local training time, it may be worth adding one or two sentences explaining why this additional cost remains acceptable in typical cross-silo federated learning scenarios, where communication is often the dominant bottleneck.

 E) The experimental protocol is generally well described. But it would improve reproducibility if the authors briefly clarified whether the reported results correspond to a single training run or to averages over multiple random seeds. If only one run was performed, simply stating this explicitly would increase transparency.

 F) The assumptions underlying the Mahalanobis-based detector are clearly presented. Nevertheless, it could strengthen the discussion if the authors briefly acknowledged that real model-update distributions are not necessarily Gaussian and that, despite this approximation, the proposed detector performs well in practice. A short remark in the limitations section would probably be sufficient.

 G) The Discussion section could be slightly enriched by commenting on situations in which RAFA may provide more limited benefits, for example when client distributions remain highly stable throughout training or when nearly all clients experience similar distribution shifts simultaneously. Including this reflection would further demonstrate the applicability and scope of the proposed method.

 H) The work is generally well written. There are only a few minor stylistic details that could improve readability, such as shortening some long paragraphs in the Introduction, maintaining a consistent notation for algorithm names (e.g., FedAvg versus FEDAVG), and being slightly more cautious with expressions such as "state-of-the-art" unless they are directly supported by comparative evidence.

Is the study design appropriate and does the work have academic merit?

Yes

Is the work clearly and accurately presented and does it cite the current literature?

Yes

If applicable, is the statistical analysis and its interpretation appropriate?

Partly

Are all the source data underlying the results available to ensure full reproducibility?

Partly

Are the conclusions drawn adequately supported by the results?

Yes

Are sufficient details of methods and analysis provided to allow replication by others?

Partly

Reviewer Expertise:

My research expertise includes intelligent engineering systems, Internet of Things (IoT), distributed systems, industrial digitalisation, embedded systems, and power electronics. I have extensive experience in the design and experimental evaluation of computational methods and engineering solutions, with particular interest in data-driven approaches and intelligent systems. For this review, my assessment focuses on the methodological quality of the proposed approach, the experimental design, reproducibility, and the engineering relevance of the contribution.

I confirm that I have read this submission and believe that I have an appropriate level of expertise to confirm that it is of an acceptable scientific standard, however I have significant reservations, as outlined above.
